# Early phase clinical trials in oncology: realising the potential of seamless designs

**DOI:** 10.1016/j.ejca.2023.05.005

**Published:** 2023-08

**Authors:** Thomas Jaki, Abigail Burdon, Xijin Chen, Pavel Mozgunov, Haiyan Zheng, Richard Baird

**Affiliations:** 1 MRC Biostatistics Unit, University of Cambridge, UK; 2 University of Regensburg, Germany; 3 Department of Oncology, University of Cambridge, UK

**Keywords:** Efficient trials, Model-based designs, Dose-determination

## Abstract

**Background:**

The pharmaceutical industry’s productivity has been declining over the last two decades and high attrition rates and reduced regulatory approvals are being seen. The development of oncology drugs is particularly challenging with low rates of approval for novel treatments when compared with other therapeutic areas. Reliably establishing the potential of a novel treatment and the corresponding optimal dosage is a key component to ensure efficient overall development. A growing interest lies in terminating developments of poor treatments quickly while enabling accelerated development for highly promising interventions.

**Methods:**

One approach to reliably establish the optimal dosage and the potential of a novel treatment and thereby improve efficiency in the drug development pathway is the use of novel statistical designs that make efficient use of the data collected.

**Results:**

In this paper we discuss different (seamless) strategies for early oncology development and illustrate their strengths and weaknesses through real trial examples. We provide some directions for good practices in early oncology development, discuss frequently seen missed opportunities for improved efficiency and some future opportunities that have yet to fully develop their potential in early oncology treatment development.

**Discussion:**

Modern methods for dose-finding have the potential to shorten and improve dose-finding and only small changes to current approaches are required to realize this potential.

## 1 Introduction

It is well-recognized drug development is risky with a very high rate of attrition (Wong et al., 2019). The development of oncology drugs is particularly challenging with low rates of approval for novel treatments when compared with other therapeutic areas (Honkala et al., 2022). To accelerate the development and approval of oncology drugs with high clinical effectiveness, cost-effectiveness, and low attrition rate in the future, the US Food and Drug Administration (FDA) promotes innovation and modern technology to overcome corresponding challenges (Spreafico et al., 2021) and only recently started to Project Optimus^1^ in order to reform dose optimization and dose selection.

One important aspect of innovation in drug development are adaptive designs, in which seamless phase I/II designs are a particularly critical application in early phases of modern drug development (Pallmann et al., 2018, Mozgunov & Jaki, 2019). These seamless designs seek to establish the safe range of doses while also learning about the potential activity of a novel agent within a single study that forms the basis of identifying the best dose to use in later development. As a critical component of the expensive, time-consuming drug development pipeline, clinical trials are traditionally divided into sequential, separate phases, where drugs are evaluated for safety in phase I, early signals of efficacy in phase II, and then investigated against standard of care in large randomised phase III clinical trials. Decreasing the high attrition rates in drug development continues to be a primary challenge for the pharmaceutical industry. One of the main challenges in achieving this goal is to strike an appropriate balance between drug safety and efficacy particularly in the early-phase studies (i.e., phase I and phase II trials, Khanna, 2012; Bowes et al. 2012; Waring et al. 2015).

Phase I clinical trials in oncology, wherein a new drug or drug combination is applied for the first time to humans, are the foundation of a successful clinical drug development programme. These studies are crucial because they define the drug dose and schedule to be further evaluated in subsequent trials. Conventionally, there is an underlying assumption that the dosage of a drug is related to undesirable (toxic) outcomes and that higher toxicity implies higher activity. This relationship could be assumed as monotonic (Rosenberger and Haines, 2002) or non-monotonic (Conolly and Lutz, 2004; Bretz et al., 2008). Most methods for phase I trials are based on the assumption of monotonicity; namely, the probability of a dose-limiting toxicity (DLT) occurring in a patient is monotonically increasing with dose (O’Quigley, Iasonos, and Bornkamp, 2017). Consequently, toxicity has traditionally and most widely been used as the primary endpoint for phase I trials evaluating cytotoxic agents. In light of the risk of severe side effects, such trials are performed as dose-escalation studies, wherein the dose of an investigational treatment is levelled up or down based on the DLT assessment from patients already treated. To target the primary objective of identifying the maximum tolerated dose (MTD) for cytotoxic therapies in oncology, a large number of dose-escalation approaches have been proposed: algorithmic approaches (including the popular 3 + 3 design, Storer, 1989), model-based designs (e.g. O’Quigley, Pepe, and Fisher, 1990) and curve-free methods (e.g. Gasparini and Eisele; 2000). A comparison of these classes of trial designs can be found in Jaki et al. (2013). Note that the approaches developed during the era of cytotoxic agents assume that both toxicity and activity increase with higher doses.

With the emergence of molecularly targeted anticancer agents, alternative endpoints to define optimal biological activity are starting to be used more commonly. Specifically, pharmacokinetic and pharmacodynamic effects, such as plasma drug concentration and target inhibition in tumour or surrogate tissues, have been advised as an alternative primary endpoint besides toxicity (Le Tourneau et al, 2009). As a result, a shift from traditional phase I designs to innovative seamless phase I/II designs, with added flexibility and special considerations, is currently ongoing. Even though it has been demonstrated that seamless designs are an efficient and flexible approach in simulation studies and some examples of recent applications of seamless phase I/II trials are available (e.g. Hobbs et al., 2019), the uptake of seamless phase I/II trials in clinical practice is still slow (Jaki, 2013; Love et al., 2017). In this work we seek to provide some insights in the benefits of modern seamless trial methodology and discuss key aspects based on real world examples.

## 2 Traditional dose-finding gone wrong

Despite all efforts to ensure that the “best” dose is identified during a Phase I trial, sometimes this goal is not achieved. The example of fulvestrant is one such situation, highlighting that rational and rigorous dose-finding studies are required to avoid large phase III registration trials with a wrong or a suboptimal dose.

The Phase I clinical trial of fulvestrant demonstrated that a short-acting formulation of fulvestrant administered daily for 7 days before primary breast surgery was well tolerated and had antiestrogenic and antiproliferative effects (DeFriend et al., 1994). Fulvestrant has very poor oral bioavailability so is administered as an intramuscular (i.m.) injection. A Phase II study with the current long-acting formulation, which was administered once monthly, showed that fulvestrant was effective in women who had breast carcinoma that progressed after tamoxifen therapy (Howell et al., 1996). In the Phase II trial, there were two long-term dose levels for 19 postmenopausal patients with tamoxifen-resistant advanced breast cancer enrolled. For appraisal of drug safety, patients 1-4 received escalating doses of fulvestrant, starting with 100 mg in the first month and increasing to 250 mg i.m. from the second month onwards, following confirmation of lack of local or systemic drug toxicity at the 100 mg dose. Patients 5-19 received 250 mg/m i.m. from the onset. The results demonstrated a clinical benefit, no serious drug-related and no effect on the pharmacokinetic results, illustrating the clinical activity and tolerability of the fulvestrant at the 250 mg/m dose regimen.

Initially, fulvestrant was approved at a single 250 mg i.m. injection per month as a second-line therapy after progression/relapse on antiestrogen therapy. Its clinical efficacy was established in two phase III registration trials (Howell et al., 2002; Osborne et al., 2002), for postmenopausal women with advanced breast cancer. Whilst 250 mg/month of fulvestrant yielded some clinical efficacy, experience and pharmacokinetic and clinical data from phase III trials had led to speculation that there may be scope to improve its clinical efficacy with higher-dose (HD) regimens based on evidence of dose-dependent effect (Robertson et al., 2001; Robertson, 2007). Subsequently, the recommended dose was revised to 500 mg/m following gains in efficacy with improved progression-free survival, based on evidence from the phase III Comparison of Faslodex in Recurrent or Metastatic Breast Cancer (CONFIRM, NCT00099437, Leo et al., 2014).

The dose-response story of fulvestrant indicates that rigorous and reliable approaches are required to take all available information into account to guide dose-finding studies and to improve the success rate of Phase III clinical trials. In the case of fulvestrant determination of the optimal dose might have been improved by using two different ideas (ideally in conjunction):

i by using modern dose-finding methods during the phase I study (see section 3)
ii by seamlessly assessing safety and activity in one study (see section 4).

## 3 Modern model-based dose-finding based on toxicity

Following the seminal work on the continual reassessment method (CRM, O’Quigley et al., 1990), model-based designs have gained increasing popularity for planning phase I dose-finding trials (Love et al., 2017, Wheeler et al., 2019). Such designs have the major advantage of estimating the dose-toxicity relationship based on a statistical model, fitted to the trial data that have been accrued on all doses investigated thus far. The risk of toxicity per dose can then be accurately assessed and, as a consequence, patients have a better chance of receiving a dose that is most likely to be the MTD (Iasonos et al., 2008). In a sequential phase I trial, the dose-toxicity relationship can be re-estimated by the inclusion of data from a new patient cohort. The consequence of using a typical model-based design in phase I trials is generally not a shortening of the study duration (or the number of patients required), but for more accurate estimation of the MTD. The result of this has been shown to yield improved success rates of development programmes as a whole (Conaway & Petroni, 2019). Despite this, there is room for further improvement to find an *optimal* dosage of a new treatment, that is the dose with the best safety/activity trade-off, in early drug development and model-based methods can readily be extended for this purpose.

In the above and throughout this manuscript, we discuss ideas and principles on the basis of single-agent dose-escalation trials for ease of description and discussion. The general concepts do, however, also extend to dose-combination studies and schedule-finding studies.

## 4 How to seamlessly assess safety and efficacy under one protocol

In light of the changing landscape of oncology drugs away from cytotoxic drugs, emphasis is now moving away from identifying the MTD but instead to determine the *optimal biologic dose* (*OBD*) which is viewed as the dose that (optimally) balances the risks of toxicity with the chance of seeing activity of the drug. Studies that seek to find this balance are called seamless Phase I/II trials and are characterised by the aim to identify the optimal dose, as well as the schedule, for late stage (Phase III) assessment within a single study protocol. We have identified three different types of seamless Phase I/II trials, namely:

- Dose-escalation followed by ad-hoc expansion cohort(s)
- Dose-escalation followed by expansion cohort(s) informed by statistical considerations
- Dose-determination with simultaneous evaluation of toxicity and efficacy

The first of these is nowadays frequently used in some centers and could therefore be viewed as a traditional approach. We nevertheless included this approach here as it combines the Phase I (safety) and Phase II (activity) objectives in one protocol and hence is a seamless trial. Since data from Phase I do not contribute to the activity assessment and similarly Phase II data do not contribute to dose-identification, however, it will not lead to improved identification of the OBD, but might shorten the development timelines compared to running two separate studies. Below we describe them on the basis of three examples and discuss their merits and demerits.

### Seamless Phase I/II trial with an ad-hoc cohort expansion

Dika et.al (2019) conducted a dose-escalation study with an expansion cohort in advanced hepatocellular carcinoma. The objective of the trial was to identify the MTD among three dose levels (0.30mg/kg, 0.60mg/kg, 0.75mg/kg) and study its activity in a larger group of patients. The target toxicity level, i.e. the maximum acceptable risk of toxicity, was however not explicitly specified. The 3+3 design (Storer, 1989) was used for the dose-escalation part and no DLTs were observed at any of the dosing levels (zero out of 12 patients in Phase I) − Table 1. The highest dose, 0.75 mg/kg, was consequently declared as the MTD. A further 31 subjects were planned to be enrolled at the declared MTD. However, after the development of grade 4 thrombocytopenia in two subjects during the expansion phase, the MTD was revised to a reduced dose of 0.6 mg/kg.

At the time when the safety signal was identified, 16 patients had their treatment already started. The dose for these patients was subsequently de-escalated for later treatment cycles (Table 1). These were not used in the formal safety analysis. Such a large expansion based on limited data resulted in little learning about the newly declared MTD − only four additional patients were recruited on the declared MTD, 0.60mg/kg during the expansion phase.

Data at the end of the expansion phase might suggest that the MTD could have been reconsidered given 1/9 DLTs on 0.60mg/kg (declared MTD) and 0/16 DLTs in the patients who switched to a lower dose. This would depend on the target toxicity level − for example, for a target toxicity level of 30%, one could have concluded that 0.60 mg/kg is underdosing. Finally, as the rule-based 3+3 design was used, the trial missed the opportunity to increase the efficiency through borrowing information across doses (e.g. the more drug the more toxicity risk) and to account for the data in patients with “switched treatment”. A further potential criticism of this design is that the size of the expansion cohort was not based on formal assessment of activity considerations, so that it is unclear if the study had sufficient power to show activity if present.

### Seamless Phase I/II trial with 3+3 design followed by Simon’s design

The Phase I/II study of dasatinib in relapsed or refractory Non-Hodgkin lymphoma (NCT00550615) reported in Umakanthan et al. (2019) utilized a seamless design that combined a traditional 3+3 dose-escalation design with a non-randomized Simon’s design in order to evaluate the safety and efficacy of dasatinib. Three dose levels, 100, 150 and 200 mg of dasatinib were investigated and the main objective of the study was to determine the MTD and to assess the objective response rate. The dose found to be the most tolerable and also has the best activity outcomes during the dose-escalation part, was to be evaluated in the second part of the study. For the latter, initially 10 patients were to be recruited and if two or more responded 19 more patients were to be enrolled further.

During the dose-escalation phase, the study sequentially allocated 14 patients: three patients received 100 mg, three patients received 150 mg, and eight patients received 200 mg of dasatinib daily. The MTD was determined to be 200 mg as no DLTs were observed. During the initial stage of the Phase II evaluation an increased rate of grade 3 pleural effusions was noted so that the dose in the phase II portion was reduced to 150 mg for the remaining patients in the study. A total of 19 patients were recruited during this part of the study, with 10 patients receiving 200 mg and 9 receiving 150 mg. As a consequence of the dose reduction during the phase II evaluation, further activity appeared to no longer follow the pre-planned Simon’s design and instead overall response assessment across all patients have been reported.

Amongst all patients in whom response assessment was possible (24 of 33 patients enrolled both during the dose-escalation and expansion phase) the objective response rate was 29% (7/24) with a 95% confidence interval (CI) of (13%, 51%). The clinical benefit rate was 71% (17/24) with a 95% CI of (49%, 87%).

As in the previous example, the use of the 3+3 design meant that there was no formal opportunity to continuously learn from accumulating safety data in the study, a feature that might have been helpful given the safety signals observed during the Phase II evaluation.

### Seamless Phase I/II with simultaneous evaluation of toxicity and efficacy

The Matchpoint trial (ISRCTN98986889, Copland et al., 2019) was a seamless phase I/II dose finding study, aiming to estimate a tolerable dose that can yield the greatest efficacy of ponatinib, in combination with conventional chemotherapy (FLAG-IDA regimen), for treating patients in the blastic transformation phase of chronic myeloid leukaemia (CML). Four dose levels of ponatinib, 7.5, 15, 30, 45 mg/day, were available for exploration in the study. The study used toxicity during the first cycle of treatment, together with haematologic or cytogenetic response at the end of the first cycle as a measure of activity, to inform dose-escalation decisions. In particular the so-called EffTox design (Thall & Cook, 2004) was used to define the trade-off between safety information and activity information.

A total of 17 patients were enrolled in successive cohorts, each contributing both safety and activity information towards the decision about subsequent cohorts dose and the final dose recommendation. The dose of 30 mg/day, which is also the starting dose, was shown to provide the best safety/activity trade-off. Hence this dose was recommended at the end of the trial as a tolerable combination in blast phase CML, with promising activity.

Brock et al. (2018) reviewed several practical challenges of the EffTox design, when implemented in the Matchpoint trial, along with their solutions. Notably, the Matchpoint trial was one of the few published examples of seamless phase I/II studies that had utilised both toxicity and activity information for interim dose selections based on a Bayesian probabilistic model.

A strength of the design is that all data contribute to the safety and activity assessment in a formal way. As part of the design the current dose is chosen to hone in on the optimal dose, meaning that exploration of uninteresting doses (e.g. doses that are more toxic but provide limited added activity) is reduced. This strength, however, comes at the cost of more complex statistical modelling and significant amount of additional planning time required to ensure that the design and trade-off between safety and activity is fit for purpose.

### Reflections on seamless trial examples

While the approaches above are commonly referred to in the literature as Phase I/II trials, they are different in both the decision-making process and the information provided in the end.

In the Phase I trial followed by a cohort expansion, formal decisions during the doseescalation part of the trial are made on the toxicity/tolerability information only. Looking at the safety data only can facilitate more rapid decision-making as the safety endpoints typically have shorter evaluation windows than activity/efficacy endpoints, and is more straightforward as only one source of information is used. Moreover, both Phase I and Phase II parts of the trial are conducted using the same infrastructure (most of which would be set up for a single trial) and often the same facilities. This can provide further logistical gains to reaching a conclusion faster.

This approach can be efficient in terms of collecting more toxicity and activity data on the recommended MTD, as the whole expansion proceeds at a single dose level and the end data are less sparse (and are indeed about the dose of most interest). This can provide good evidence to go into further investigations of this dose. However, this efficiency is subject to expanding at the truly MTD, which is often unlikely, due to the limited data made for the expansion decision. For example, in the advanced hepatocellular carcinoma trial (Dika et al., 2019), the expansion was made on a dose that was later declared as overly toxic. A large expansion on a single dose based on limited data may result in quite restricted learning about the new declared MTD. Using a smaller cohort size for the expansion phase, or continuing the dose-escalation trial for an equivalent total sample size, is likely to facilitate a more ethical and statistically efficient learning about the toxicity. It is more ethical because fewer patients are assigned possibly more toxic doses (and hence potentially avoiding some DLTs). It is meanwhile more statistically efficient for assigning more patients close to the finally recommended MTD that supports a better efficient learning about its toxicity.

Another crucial aspect of this type of trial is the size of the expansion and the decisionmaking at its end. In such an expansion, there is typically no formal criteria for both. This leads to further inefficiencies in such trials. Without a formal sample size calculation for the expansion cohort(s), it is unclear what effects on the activity can be claimed as clinically meaningful. Moreover, in the absence of a clear decision-making framework, the probability of making incorrect conclusions is also unclear. Both can jeopardise the value of the trial and its acceptability by wider groups.

The design as given in the example in Umakanthan et al. (2019) with Simon’s two-stage design in the expansion phase, can resolve the latter concerns about the sample size and decision-making. It still facilitates more rapid decision-making in the drug development process through setting up a single infrastructure for both phases. In this sense, such a setting would represent two separately planned trials but run seamlessly for the logistical gains. However, such operationally seamless trials could still suffer from expanding to a large group of patients at once based on the limited data. Simon’s two-stage design, for example, accounts for the efficacy information only in the decision-making yet does not formally include the toxicity assessment. Were the recommended Phase I dose to be considered overly toxic, and a new MTD to be declared during (or after) the Phase II part, there would be limited learning about it due to exhausting all the resources for the toxic dose.

A Phase I/II design which analyses both toxicity and efficacy endpoints simultaneously, tackles this deficiency of the cohort expansion designs while maintaining the operational gains of conducting initial safety and efficacy assessments within one established infrastructure. By allowing dose change throughout the whole trial, such Phase I/II trials mitigate the risk of assigning many patients to overly toxic doses, and hence can be considered as a more ethical approach to first-in-patient Phase I studies. Furthermore, by assessing both safety and efficacy, a typical recommendation of such a trial will be the optimal biological dose (OBD), a dose that balances the risks and benefits. While such a trial can be expected to result in fewer patients at the recommended OBD (compared to the expansion), more patients would be treated in the neighbourhood of the OBD that, again, contributes toward focusing on a single possibly incorrect dose. Additionally, by allowing a dose change throughout the trial and assessing both endpoints on them, such trials can provide richer data to establish the dose-toxicity and dose-efficacy relationships of the studied drug. This can facilitate a more efficient recommendation of a dose (or doses) for further evaluation rather than recommending (or not recommending) the one at which the expansion was made.

This Phase I/II seamless design approach, however, can be associated with several challenges which are, primarily, logistical. Identification of the OBD requires tracking of two (or more) outcomes throughout the trial that can take more time than when assessing toxicity only. In a multi-site trial, it should also be ensured that all sites have resources to evaluate both outcomes. Furthermore, there might be different evaluation windows for both endpoints with the efficacy window, typically, being longer. While waiting for the complete evaluation can significantly increase the trial duration, there are approaches allowing only partial evaluation of the response (Cheung & Chappell, 2000) but still leading to efficient decision-making. Finally, such a design typically has no formal hypothesis testing as it focuses on the selection of the OBD.

## 5 Challenges and opportunities for seamless trials

In this section, we reflect on the different approaches to seamless Phase I/II designs and specifically consider good practice, the often missed and future opportunities to improve early phase oncology trials.

### Good practice

One principal feature of seamless clinical trial designs is the efficient use of all available data for decision making. As a first step, it means that model-based and model-assisted designs should be routinely used instead of algorithmic approaches such as the 3+3 design. In phase I/II dose-finding trials, it is strongly advisable to collect data on both toxicity and efficacy outcomes from each patient throughout the trial and incorporate them in the decision making throughout. Conducting the trial first with a dose-escalation procedure before an embedded proof-of-concept study may sometimes be desirable. In those circumstances, however, it is essential to continue monitoring toxicity data at the individual level until trial completion. When the dose selection decisions in the phase I component are based on toxicity data only, collecting efficacy data from this stage can still be advantageous. Because through combining these data with the newly accrued efficacy data from the phase II component, higher statistical power to test the treatment effect and a better characterisation of the dose-efficacy relationship is possible.

Full specification of the decision criteria, including the dose (de-)escalation rules, when and how to expand on certain selected dose(s), etc., should be precisely outlined in the protocol. Any unplanned decision making may induce bias and possibly yield a suboptimal dose for further evaluation in a confirmatory setting. Moreover, the size of the expansion cohort(s) should be based on statistical considerations to ensure that any findings can be meaningfully interpreted. This also means that the method for determining the sample size of the expansion cohort(s) should be provided in the protocol. As noted earlier, expansion on several doses rather than a single dose can be more ethical and statistically efficient, since the MTD may be incorrectly selected from the dose-escalation procedure based on limited information - a problem observed in two of our examples.

### Frequently missed opportunities

Above we have made the argument that collecting both safety and efficacy data throughout the study and incorporating activity data from the Phase I dose-finding component in the Phase II evaluation is efficient, even when only considering a single selected dose to explore further. Similar to the use of dose-toxicity models that govern the dose-escalation, dose-activity models can be used to obtain a better understanding of the activity profile and hence help to identify the dose with the best trade-off between safety and activity. This would be particularly relevant, when multiple doses are expanded.

Similarly, using a model to describe the dose-toxicity relationship means that the model can continue to be updated once the Phase II part has been initiated. This has the advantage that learning about the MTD continues throughout the study so that necessary changes to the MTD can be identified based on the same coherent framework. In addition, it would often be more quickly than when relying on ad-hoc adjustments and replaces the need for safety run-ins at Phase II trials. In this case it is helpful to have pre-determined rules to guide the continued safety monitoring in a similar fashion as is routinely done during the Phase I portion of the study. For example, one can use the same rule to determine the MTD as during the Phase I portion (e.g., the dose whose probability of having a toxicity risk between 25 and 35 % is largest), but only make a dose change after at least 5 patients have been recruited in the Phase II portion of the study to avoid changing the dose to quickly. Alternatively, a minimum difference of 5% in the estimated toxicity risk between the dose used in the Phase II portion and the MTD according to the model could be used.

Another area of often missed opportunity relates to the utility of pharmacokinetic (PK) information. It is sometimes argued that the dose of a treatment is meaningless on its own right, as what reaches the relevant site is important. PK information often plays a secondary role in dose-escalation decisions. This is often due to the late availability of PK information on the current cohort. More precisely, due to a lack of resources and/or the time required to analyse the samples, PK information would likely be collected and interpreted only after the next dose-escalation decision has been made. When this hurdle can be overcome, however, PK information can be incorporated directly in the dose selection decision in real time (e.g. Cotterill et al., 2015) or alternatively exposure-toxicity/exposure-activity (e.g. Micallef et al., 2022) can be considered directly instead of dose-toxicity/dose-activity.

### Seamless Phase I/II trial with 3+3 design followed by Simon’s design revisited

**Figure 1:**
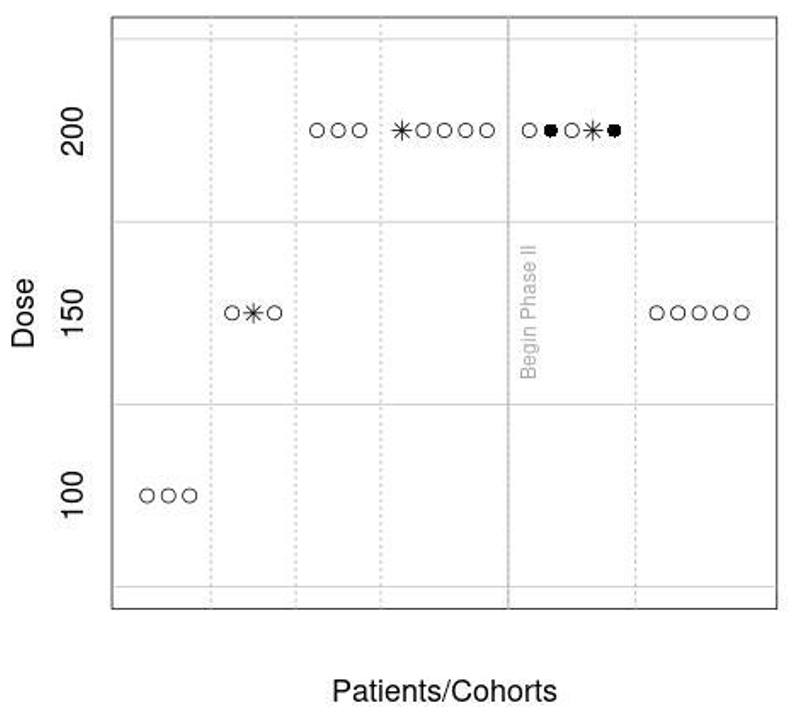
Illustration of dose allocation in dasatinib study if the EffTox approach had been used. Open circles correspond to patients without toxicity, closed circles are for patients with toxicity and stars correspond to patients that responded.

To illustrate the potential benefit of simultaneously assessing safety and efficacy we revisit the Phase I/II study of dasatinib (NCT00550615) reported in Umakanthan et al. (2019). In this illustration we use the EffTox design by Thall & Cook (2004) instead of the original design and that a toxicity risk of at most 15% is deemed acceptable since observing 2 grade 3 pleural effusions among 10 patients triggered a dose reduction. Figure 1 shows the allocation of patients for the first 24 patients and assumes that safety is revisited in the Phase II portion after every 5 patients. For this sequence of observations we can see that a dose reduction is already recommended by the model after the first 5 patients in the Phase II portion. Note that we did not have the information on which patient had a DLT or response and hence randomly generated when toxicities and activity was observed in line with the frequencies reported for the trial. Different sequences of observations might, however, result in different recommendations.

### Future opportunities

One of the most exciting future opportunities for seamless Phase I/II trials in oncology is the use of novel biomarkers such as circulating tumour DNA (ctDNA) and novel imaging techniques which are promising tools to identify if a patient reacts favourably (or insufficiently) to a treatment much faster than traditional metrics such as progression-free survival. Such biomarker data can then be used to either determine sufficient activity (e.g. favourable ctDNA decrease) or lack thereof (e.g. no decrease in ctDNA levels after initial treatment period) quickly enabling these activity data to be readily available for dose selection decisions during the escalation phase. A challenge to embrace these future opportunities is the limited availability of methods to exploit these biomarkers at present and hence future work in this area is required.

Similarly, use of PK-PD models to guide dose-escalation decisions has great promise. Due to the challenges in obtaining PK data sufficiently quickly (as outlined above) and the time required to build rather complex models, this promise is yet to be realised. One possible way forward could be to utilise PK-PD models that have been established for similar compounds (e.g. with the same mechanism of action) in the first instance and consecutively refine these models as data on the novel compound are observed. In this case one could well imagine the use of PK-PD models linked to multiple PD makers of interest.

Finally, an often overlooked aspect in Phase I/II dose-escalation trials is patient heterogeneity. Some statistical methods to account for subgroups have been developed; see Cotterill & Jaki (2018) for example. These often rely on subgroups defined through a single biomarker-defined subgroup. The hope is that novel methods that allow personalization of the optimal dose during the Phase I/II trial can be developed. This is not only to ensure appropriate dosing would be used in the confirmatory trials and beyond, but also that participation in the study maximises the patients benefit.

## Figures and Tables

**Table 1:** Number of patients assigned to each of the dose and number of DLTs observed after Phase I and after the expansion cohort part. The column “0.75 mg/kg -> 0.60 mg/kg” corresponds to the patients who have switched the dosing level part through the treatment.

Dose Levels	0.30 mg/kg	0.60 mg/kg	0.75 mg/kg	0.75 mg/kg -> 0.60 mg/kg
After Phase I	0/3	0/5	0/4	NA
After Expansion	0/3	1/9	4/15	0/16

## Highlights

- Modern methods for dose-finding have the potential to improve dose-selection
- Seamless Phase I/II trials are efficient in determining a safe and active dose
- Small changes to current approaches can improve efficiency of dose-finding trials

## Footnotes

1
https://www.fda.gov/about-fda/oncology-center-excellence/project-optimus
